# Author Correction: Impact of commonly administered drugs on the progression of spinal cord injury: a systematic review

**DOI:** 10.1038/s43856-025-01345-0

**Published:** 2025-12-30

**Authors:** Lucie Bourguignon, Louis P. Lukas, Bethany R. Kondiles, Bobo Tong, Jaimie J. Lee, Tomás Gomes, Wolfram Tetzlaff, John L. K. Kramer, Matthias Walter, Catherine R. Jutzeler

**Affiliations:** 1 Department of Health Sciences and Technology, ETH Zurich, Zurich, Switzerland; 2 SIB Swiss Institute of Bioinformatics, Lausanne, Switzerland; 3 Schulthess Klinik, Zurich, Switzerland; 4 International Collaboration on Repair Discoveries (ICORD), University of British Columbia, Vancouver, BC Canada; 5 Department of Zoology, University of British Columbia, Vancouver, BC Canada; 6 Department of Biosystems Science and Engineering, ETH Zurich, Basel, Switzerland; 7 Djavad Mowafaghian Centre for Brain Health, University of British Columbia, Vancouver, BC Canada; 8 Department of Anesthesiology, Pharmacology, and Therapeutics, Faculty of Medicine, University of British Columbia, Vancouver, BC Canada; 9 Department of Urology, University Hospital Basel, University of Basel, Basel, Switzerland

**Keywords:** Spinal cord diseases, Spinal cord injury

Correction to: *Communications Medicine* 10.1038/s43856-024-00638-0, published online 24 October 2024

In the version of the article initially published, there were labelling errors in Fig. 7d, where the order of studies on the left-hand side was incorrect, with Leech et al. (2014) appearing in the top row rather than second from bottom, while the year listed for Aminmansour et al. (2016) was incorrectly listed as 2012. The figure is now amended in the HTML and PDF versions of the article.

