# Supplementary material for: Impact of commonly administered drugs on the progression of spinal cord injury: a systematic review
Source: Commun Med (Lond). 2024 Oct 24;4:213. doi: 10.1038/s43856-024-00638-0 (PMC11502874; doi:10.1038/s43856-024-00638-0)
Supplement: Supplementary file 2 — Supplementary Information [file 43856_2024_638_MOESM2_ESM.pdf]

# **Supplementary Material** for **Impact of commonly administered drugs on the progression of spinal cord injury: a systematic review**

Lucie Bourguignon [1,2,3*#], Louis P. Lukas [1,2,3*#], Bethany R. Kondiles [4,5], Bobo Tong [4], Jaimie J. Lee [4], Tomás Gomes [6], Wolfram Tetzlaff [4], John L.K. Kramer [4,7,8], Matthias Walter [9], Catherine R. Jutzeler [1,2,3]

[1]*Department of Health Sciences and Technology, ETH Zurich, Zurich, Switzerland* (Lucie Bourguignon; Louis P. Lukas; Catherine R. Jutzeler)
[2]*SIB Swiss Institute of Bioinformatics, Lausanne, Switzerland* (Lucie Bourguignon; Louis P. Lukas; Catherine R. Jutzeler)
[3]*Schulthess Klinik, Zurich, Switzerland* (Lucie Bourguignon; Louis P. Lukas;Catherine R. Jutzeler)
[4]*International Collaboration on Repair Discoveries (ICORD), University of British Columbia, Vancouver, British Columbia, Canada* (Bethany R. Kondiles; Bobo Tong; Jaimie J. Lee; Wolfram Tetzlaff; John L.K. Kramer)
[5]*Department of Zoology, University of British Columbia, Vancouver, British Columbia, Canada* (Bethany R. Kondiles, PhD)
[6]*Department of Biosystems Science and Engineering, ETH Zurich, Basel, Switzerland* (Tomas Gomes)
[7]*Djavad Mowafaghian Centre for Brain Health, University of British Columbia, Vancouver, British Columbia, Canada* (John L.K. Kramer)
[8]*Department of Anesthesiology, Pharmacology, and Therapeutics, Faculty of Medicine, University of British Columbia, Vancouver, British Columbia, Canada* (John L.K. Kramer)
[9]*University Hospital Basel, University of Basel, Basel, Switzerland* (Matthias Walter, MD, PhD)

[*]These authors contributed equally

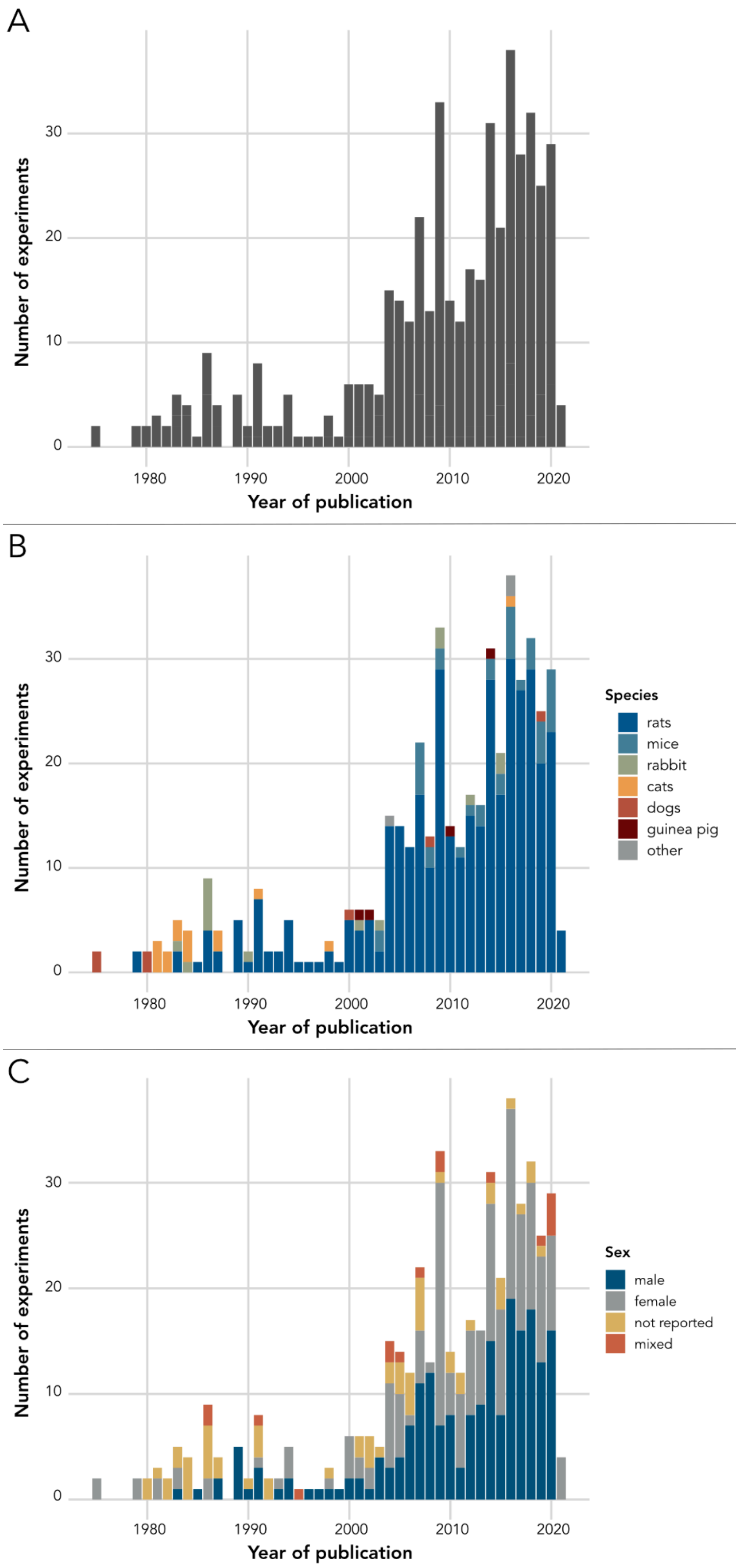


**Supplementary Figure S1. Publication trends over time. A** General overview of the number of experiments included per year of publication. **B** Details of distribution of species used in animal models over time. **C** Details of distribution of sex in animal models over time.

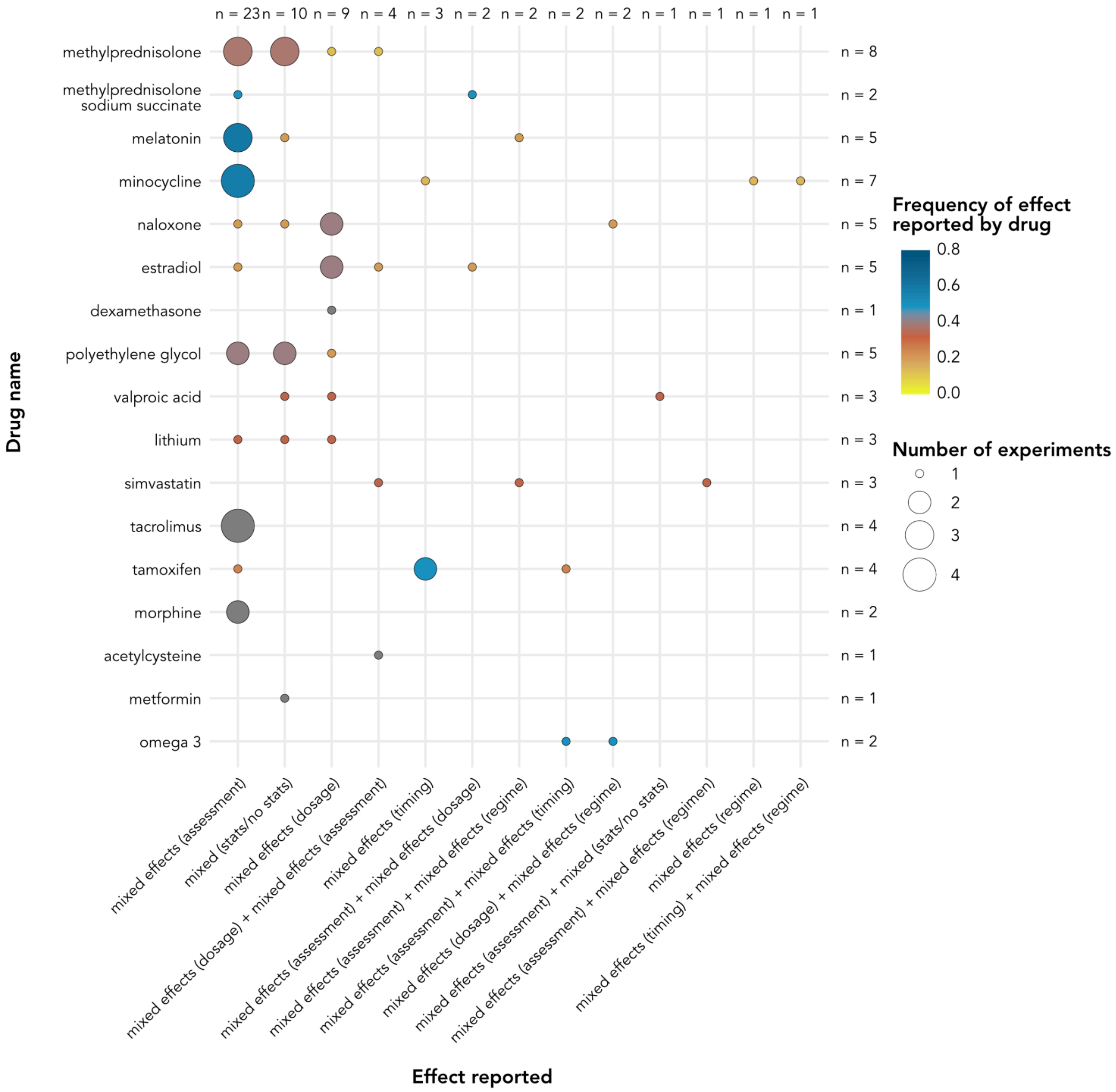


**Supplementary Figure S2. Details of mixed drug effects reported in at least five experiments.** Circle size is proportional to the number of experiments reporting the effect of interest. Circles are colored proportionally to the frequency that the effect of interest represents among all experiments studying the drug of interest.

**Supplementary Table S1.** Neurological and functional outcomes for human studies included in the review.

| Category | Assessment name reported | Assessment |
|---|---|---|
| neurological | neurological (motor and sensory) | marked recovery (combination of improvement in AIS grade and walking function) |
| | | pinprick, light touch, motor function scale |
| | | ASIA motor and sensory scores |
| | | ASIA scale: motor and sensory composites |
| | | ASIA motor score, ASIA sensory score |
| | | motor score; light touch (LT) and pin prick (PP) scores |
| | neurological (motor) | ASIA motor score |
| | | ASIA Motor score |
| | | ISNCSCI motor score |
| | | strength |
| | | discharge motor score |
| | neurological (other) | improvement to level of injury (change in segment to more caudal location) |
| | injury severity | marked recovery (combination of improvement in AIS grade and walking function) |
| | | improvement in ASIA scale |
| | | ASIA impairment score and grade |
| | | ASIA grade |
| functional | functional (general) | Spinal Cord Independence Measure |
| | | Functional Independence Measure |
| | | London Handicap scale |
| | | Short Form 36 Questionnaire |
| | | FIM discharge score |
| | functional (mobility) | FIM motor score |
| | functional (mobility and general) | Walking Index for SCI II (WISCI II), Spinal Cord Independence Measure II (SCIM II) |
| | functional (mobility and spasticity) | overground walking performance; treadmill walking performance |
| | | spastic reflexes (modified Ashworth scale); walking function |
| electrophysiology | | EMG |

**Supplementary Table S2.** Details on the bias classification for animal experiments.

| Domain of bias | Classified as “unclear risk of bias” | Classified “high risk of bias” |
|---|---|---|
| Dose | No precise dose reported, including “high dose” | Not reported |
| Species | Subspecies not reported | Not reported |
| Route | - | Not reported |
| Level of injury | No precise level or range reported, including “cervical”, “mid-thoracic, ‘thoracic”, “lumbar-sacral” | Not reported |
| Treatment time | - | Not reported |
| Results | Mixed results due to lack of statistics reported, including "mixed (stats/no stats)", "mixed effects (assessment) + mixed (stats/no stats)", "no stats" | Not reported |
| Sample size | Sample size reported as range or bounded | Not reported |
| Sex | Mixed population (male/female) with ratio not reported | Not reported |
| Blinding | Not reported | No blinding applied |
| Age | Reported as “adult”, “young” with no precise age reported | Not reported |

**Supplementary Table S3.** Reported sample sizes by species in animal studies. *Other* includes Yucatan miniature pigs (n=2) and yellow eel Anguilla anguilla L. (n=1).

| Species | Mean | SD | Median | Q1 | Q3 |
|---|---|---|---|---|---|
| cats | 26.53 | 16.50 | 24.00 | 16.50 | 31.00 |
| dogs | 33.43 | 25.13 | 26.00 | 22.00 | 32.00 |
| guinea pig | 21.00 | 7.55 | 20.00 | 17.00 | 24.50 |
| mice | 120.62 | 83.33 | 96.00 | 50.25 | 176.50 |
| other | 31.33 | 16.17 | 22.00 | 22.00 | 36.00 |
| rabbit | 69.36 | 50.57 | 47.00 | 28.50 | 133.00 |
| rats | 61.60 | 46.71 | 48.00 | 32.00 | 79.50 |
