# Supplementary material for: Impact of commonly administered drugs on the progression of spinal cord injury: a systematic review
Source: Commun Med (Lond). 2024 Oct 24;4:213. doi: 10.1038/s43856-024-00638-0 (PMC11502874; doi:10.1038/s43856-024-00638-0)
Supplement: Supplementary file 3 — Description of Additional Supplementary Files [file 43856_2024_638_MOESM3_ESM.pdf]

Description of Additional Supplementary Files

File name: Supplementary Data 1.

File description: List of drugs included in analysis. Shaded rows highlight drugs tested in combination

File name: Supplementary Data 2.

File description: Neurological and functional outcomes for animal studies included in the review

File name: Supplementary Data 3.

File description: Variables extracted from studies included for analysis

File name: Supplementary Data 4.

File description: Bias assessment by animal experiment
